# The mechanism of Renshen-Fuzi herb pair for treating heart failure—Integrating a cardiovascular pharmacological assessment with serum metabolomics

**DOI:** 10.3389/fphar.2022.995796

**Published:** 2022-12-05

**Authors:** Xiaofei Chen, Yulong Chen, Shiyang Xie, Xiaoyan Wang, Yali Wu, Hui Zhang, Ya Zhao, Jinhao Jia, Bin Wang, Weixia Li, Jinfa Tang, Xiaohe Xiao

**Affiliations:** ^1^ College of Medicine, Chengdu University of Chinese Medicine, Chengdu, China; ^2^ Henan Province Engineering Laboratory for Clinical Evaluation Technology of Chinese Medicine, Henan Province Engineering Research Center for Clinical Application, Evaluation and Transformation of Traditional Chinese Medicine, The First Affiliated Hospital of Henan University of Chinese Medicine, Zhengzhou, China; ^3^ College of Medicine, Henan University of Chinese Medicine, Zhengzhou, China; ^4^ Department of Hepatology, Fifth Medical Center of Chinese PLA General Hospital, Beijing, China

**Keywords:** Panax ginseng C.A.Mey., Aconitum carmichaeli Debeaux, heart failure, combination mechanisms, cardiovascular pharmacological, serum metabolomics

## Abstract

**Background:** Renshen-Fuzi herb pair (RS-FZ) is often used in the clinical treatment of heart failure (HF) and has a remarkable therapeutic effect. However, the mechanism of RS-FZ for treating HF remains unclear. In our study, we explored the mechanism of RS-FZ for treating HF.

**Methods:** Evaluation of RS-FZ efficacy by cardiovascular pharmacology. Moreover, Global metabolomics profiling of the serum was detected by UPLC-QTOF/MS. Multivariate statistics analyzed the specific serum metabolites and corresponding metabolic pathways. Combining serum metabolomics with network pharmacology, animal experiments screened and validated the critical targets of RS-FZ intervention in HF.

**Results:** RS-FZ significantly ameliorated myocardial fibrosis, enhanced cardiac function, and reduced the serum HF marker (brain natriuretic peptide) level in rats with HF. Through topological analysis of the “Metabolite-Target-Component” interaction network, we found that 79 compounds of RS-FZ directly regulated the downstream specific serum metabolites by acting on four critical target proteins (CYP2D6, EPHX2, MAOB, and ENPP2). The immunohistochemistry results showed that RS-FZ observably improved the expression of CYP2D6 and ENPP2 proteins while decreasing the expression of EPHX2 and MAOB proteins dramatically.

**Conclusion:** The integrated cardiovascular pharmacological assessment with serum metabolomics revealed that RS-FZ plays a crucial role in the treatment of HF by intervening in CYP2D6, EPHX2, MAOB, and ENPP2 target proteins. It provides a theoretical basis for RS-FZ for treating HF.

## Introduction

Heart failure (HF) is the final stage in the progression of most cardiovascular diseases, and it is a common, disabling, and fatal disease (Dyck et al., 2019). Currently, the primary drugs used in the clinical treatment of HF are mainly diuretics, cardiotonic, vasodilators, and angiotensin-converting enzyme inhibitors. However, improper use of these therapeutic drugs often causes side effects such as arrhythmia and ion disturbance, thus affecting the curative effect. (Zhang, 2015). Recent studies have shown that traditional Chinese medicine (TCM) treatment of HF has achieved a remarkable impact in alleviating symptoms, avoiding adverse drug reactions, and improving patients’ quality of life (Wang et al., 2017).

Renshen-Fuzi herb pair (RS-FZ) is made up of Panax ginseng C.A.Mey. (RS, Renshen in Chinese) and Aconitum carmichaeli Debeaux (FZ, Fuzi in Chinese). RS-FZ is a classic herb pair often used for HF treatment and contained in many medical preparations (Huang et al., 2020). Furthermore, a large amount of pharmacological experimental studies have shown that both RS and FZ have an independent anti-HF effect (Liu et al., 2021; Wang et al., 2021), and the therapeutic effect was enhanced after the compatibility of RS and FZ by increasing myocardial contractility, inhibiting the expression of inflammatory factors and improving hemodynamics, etc (Liu et al., 2020). However, the underlying mechanism of this herb pair is still unclear.

**FIGURE 1:**
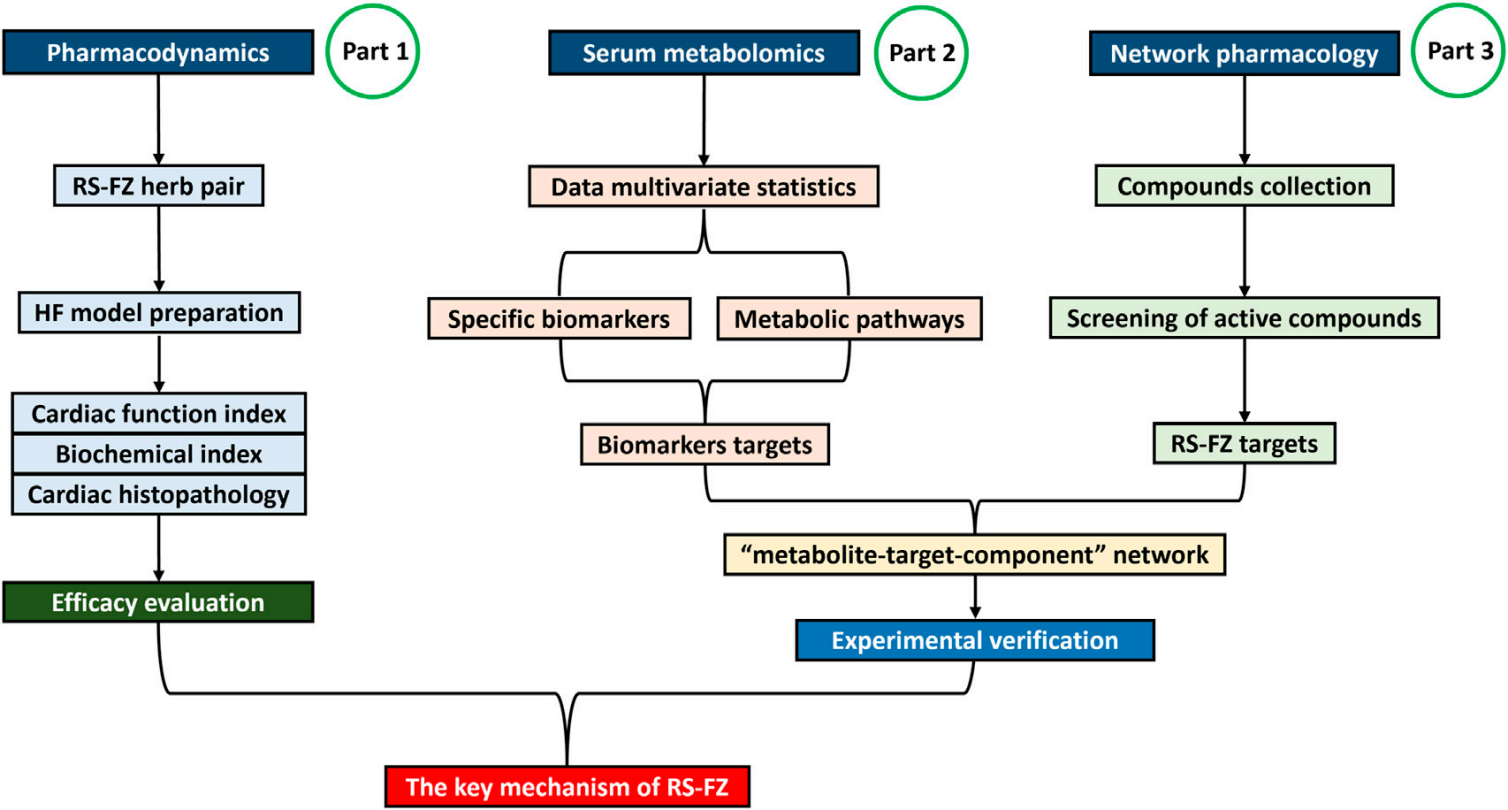
The detailed flowchart of the study design.

Through the combination of serum metabolomics, network pharmacology, molecular biology, and other systematic pharmacological research methods (Wei et al., 2018; Guo et al., 2022), the potential target of RS-FZ treatment of HF was excavated, and the molecular mechanism of RS-FZ treatment of HF was revealed. At the same time, the critical targets were verified by immunohistochemistry. This study provided a theoretical basis for the clinical application of RS-FZ (Figure 1).

## Materials and methods

### Chemicals and instruments

RS and FZ (Danfupian) were purchased from Anhui People’s Chinese Herbal Pieces Co., Ltd (Anhui, China). The source and quality of the two medicinal materials were identified according to the 2020 edition of Chinese Pharmacopoeia. Rat brain natriuretic peptide (BNP) enzyme-linked immunosorbent assay (ELISA) Kit (No. E-EL-R0126c, Elabscience). Masson staining Kit (No. 20180804). Cytochrome P450 2D6 (CYP2D6), soluble epoxide hydrolase 2 (EPHX2), Monoamine oxidase-B (MAOB), and Ectonucleotide Pyrophosphatase/Phosphodiesterase 2 (ENPP2) antibodies were purchased from Wuhan Seville Biotechnology Co., Ltd. and Wuhan Sanying Biotechnology Co., Ltd. Vivid E9 Color Doppler ultrasound diagnostic apparatus (GE Norway).

### Water extract of RS-FZ preparation

The ratio of RS-FZ (RS: FZ) in TCM prescriptions for the clinical treatment of HF was 1:2 (Luo et al., 2008). In addition, according to the 2020 edition of Chinese Pharmacopoeia, the common human doses of RS and FZ were determined to be 5 g/d and 10 g/d, respectively. RS-FZ was prepared by extracting the mixture of these two herbs twice with water for 1 h. The extract was then decanted, filtered, and dried under reduced pressure. The final yield of dry extract was 18.2%.

### Animal experimentation

Healthy SD male rats (SPF grade, 220 ± 10 g) were purchased from Beijing Weitong Lihua Experimental Animal Technology Co., Ltd. (SCXK 2016-0011). The Experimental Animal Ethics Committee approved animal feeding and experimental procedures of the First Affiliated Hospital of Henan University of Traditional Chinese Medicine (No. YFYDW2017005). After 1 week of adaptation, 30 rats were randomly divided into the sham operation (Sham) group (*n* = 8) and the myocardial infarction group (*n* = 22). According to the previous study (Zhu et al., 2015), rats prepared the myocardial infarction model by ligating the anterior descending (LAD) branch of the left coronary artery. Rats in the sham group were not ligated, and the operation was the same. The surviving rats, after modeling, were fed independently for 4 weeks. The left ventricular function of rats in the myocardial infarction group was evaluated by ultrasound, and the left ventricular ejection fraction (LVEF) < 50% was the successful preparation of the HF model (A. Chen et al., 2015).

Sixteen rats with successful modeling (Six failed in modeling) were randomly divided into the heart failure model (HF) group and RS-FZ group, with eight rats in each group. Through the dose conversion between human and rat body surface area (Reagan-Shaw et al., 2008), rats in RS-FZ group were given dried extract (0.25 g/kg/d) by gavage for 4 weeks. At the same time, Sham and HF groups received the same volume of distilled water for 4 weeks.

### Effect of RZ-FZ on cardiac function and biochemical index in rats with heart failure

Transthoracic echocardiography was used to detect and record the cardiac function of rats before, and 4 weeks after, RS-FZ was administered. From the long-axis parasternal view, the cardiac cycle was recorded by M-mode ultrasound. The rats were weighed and anesthetized with a 20% ethyl carbamate solution. The ventricular septal thickness (IVS), left ventricular end-diastolic diameter (LVESD), and left ventricular end-diastolic diameter (LVEDD) were measured by echocardiography, and the LVEF and left ventricular short-axis shortening (LVFS) were calculated. Blood was taken from the abdominal aorta and serum was centrifuged to determine the content of brain natriuretic peptide (BNP) in serum by an enzyme-linked immunosorbent assay (ELISA). Then the rats were sacrificed, and the heart was quickly removed. After removing the capsule, atrium, and right ventricular tissue, the myocardial tissue in the ischemic risk area was placed in a −80°C refrigerator and 4% paraformaldehyde for standby. Left ventricular mass index (LVMI) (‰) = left ventricular mass (mg)/body weight (g) was calculated.

### Distribution of collagen in myocardial tissue

Masson staining was used. In the same part of the left ventricle, cut about 2 mm thickness of myocardial tissue, 4% paraformaldehyde-fixed, paraffin-embedded tissue sections, staining procedures strictly following the kit instructions. Moreover, take pictures under the mirror.

### Immunohistochemistry

Tissue sections were placed in EDTA antigen repair buffer (PH9.0) for antigen repair and washed three times with PBS (PH7.4). Then they were incubated in 3% hydrogen peroxide for 25 min and washed three times with PBS. Add 3% BSA to cover the tissue evenly and block for 30 min. Add PBS to slices in a certain proportion of the first antibody (CYP2D6, EPHX2, MAOB, and ENPP2) overnight. Then the slides were washed in PBS 3 times and covered with double-antibody. DAB chromogenic agent color, washing, hematoxylin re-staining nucleus, dehydration sealing, microscopic examination. The signal of immunohistochemical staining was analyzed with Image-Pro Plus 6.0 software.

### Preparation of serum samples for metabolomics analysis

The collected rat blood was centrifuged at 4°C and 3,500 r/min for 10 min, and the serum was collected. Ultrahigh-performance liquid chromatography quadrupole time-of-flight mass spectrometry (UPLC-QTOF/MS) analyzed the serum samples after solvent extraction, protein precipitation, and high-speed centrifugation. Then, 10 μl serum samples were taken from the three groups and mixed as quality control (QC) samples to validate the stability of the UPLC-QTOF/MS system.

### Chromatography and mass spectrometry

The UPLC-QTOF/MS system comprised an Acquity Ultra-Performance Liquid Chromatography (UPLC) system and a Xevo G2-XS QTOF-MS mass spectrometer (Waters, United States). The chromatographic separation conditions are as follows. Mobile phase A was 0.1% formic acid aqueous solution, and mobile phase B was 0.1% formic acid acetonitrile solution. Gradient elution: 0–6 min, 5%–45% B; 6–8 min, 45%–75% B; 8–12 min, 75%–85% B; 12–12.5 min, 85%–100% B; 12.5–16 min, 5% B. Column temperature 40°C, flow rate 0.3 ml/min, injection volume 3 μl.

To enable high sensitivity, selectivity, speed, and precision, full information tandem mass spectrometry (MSE) technology was used in this experiment. An Electron Spray Ionization source was used for data collection in MSE continuum mode, a uniform high-frequency data acquisition mode. The accurate mass determination was performed using Leucine Enkephalin as the locking mass solution. The following electrospray source parameters were used: the electrospray capillary voltages were 2.5 kV (negative ionization mode) and 3.0 kV (positive ionization mode), the desolventizing gas temperature was 250°C, the cone gas flow was 50 L/h, the desolventizing flow was 800 L/h, the collision energy was 6 V, the mass range ranged from m/z 50-1,200.

### Data extraction and multivariate analysis

The UPLC-QTOF/MS experimental data were collected by MassLynx software (v4.1) of Waters company and imported into Progenesis QI software (v2.4) for chromatographic peak alignment, peak extraction, and normalization, and the retention time (RT) was recorded. Normalized data matrix containing sample name, RT-m/z pair, and abundance were obtained by Progenesis QI software. Then, the normalized dataset was pre-processed using Excel software based on the “80% rule” to reduce the input of missing values and to remove features with relative standard deviations (RSDs) > 30% from all the samples (Dunn et al., 2011). After editing, the normalized dataset was transformed (Log-transformation) and scaled (Pareto scaling), and analyzed by principal component analysis (PCA) and orthogonal partial least-squared discriminant analysis (OPLS-DA) using SIMCA-P 14.1 software. Variable important in the projection (VIP) and S-plot were used to predict the contribution of each data to the model. The variables with VIP >1 and |p(corr)| ≥0.5 in the OPLS-DA analysis were further evaluated with an independent sample *t*-test.

Each feature’s formula and accurate mass were submitted to ChemSpider (http://www.chemspider.com/). Furthermore, we conducted an identification analysis based on the Human Metabolome Database (https://hmdb.ca/) (Wishart et al., 2018) and Kyoto Encyclopedia of Genes and Genomes (https://www.genome.jp/kegg/) (Kanehisa and Goto, 2000) databases identification analysis. The specific metabolite with the most references was considered the terminal matching result, and the significant variables (*p* < 0.05 in ANOVA) were selected as specific metabolites were imported into the MetaboAnalyst v 5.0 online database for pathway analysis.

### Identification targets of drug and specific metabolites and network construction

The compounds of RS-FZ were searched by the Encyclopedia of Traditional Chinese Medicine database (http://www.tcmip.cn/ETCM/index.php/Home/Index/) (Xu et al., 2019) using “Ginseng” and “Aconite” as keywords. Then, the SMILES (Simplified Molecular-input line-entry system) of the compounds were found in the PubChem database (https://pubchem.ncbi.nlm.nih.gov/) (Kim et al., 2021). The SMILES were copied to SwissADME (http://www.swissadme.ch/) (Daina et al., 2017) to predict the absorption and drug-like properties of compounds. The active compounds of RS-FZ were screened with “High” GI absorption (Gastrointestinal absorption) and Druglikeness properties as indexes and supplemented with literature retrieval (Wang et al., 2016; Xiao et al., 2017; Zhang et al., 2017). The SMILES of compounds were imported into SwissTargetPrediction (http://www.swisstargetprediction.ch/) (Daina et al., 2019) with the property selected as “*Homo sapiens*” to collect compound targets. In addition, the targets of specific metabolites were collected from the MBROLE database (http://csbg.cnb.csic.es/mbrole2/) (Lopez-Ibanez et al., 2016). Finally, the “metabolite-target-component” (M-T-C) interaction network was established by Cytoscape 3.8.2 software.

### Statistical analysis

Data were analyzed by one-way analysis of variance (ANOVA) followed by a Dunnett’s test or a Kruskal–Wallis ANOVA on Ranks followed by a Dunn’s test for multiple comparisons and expressed as the means ± SD. *p* < 0.05 was considered a significant difference. SIMCA-P 14.1 software was used for pattern recognition analysis of PCA and OPLS-DA serum metabolic compounds.

## Results

### The therapeutic effect of RS-FZ for heart failure

**FIGURE 2:**
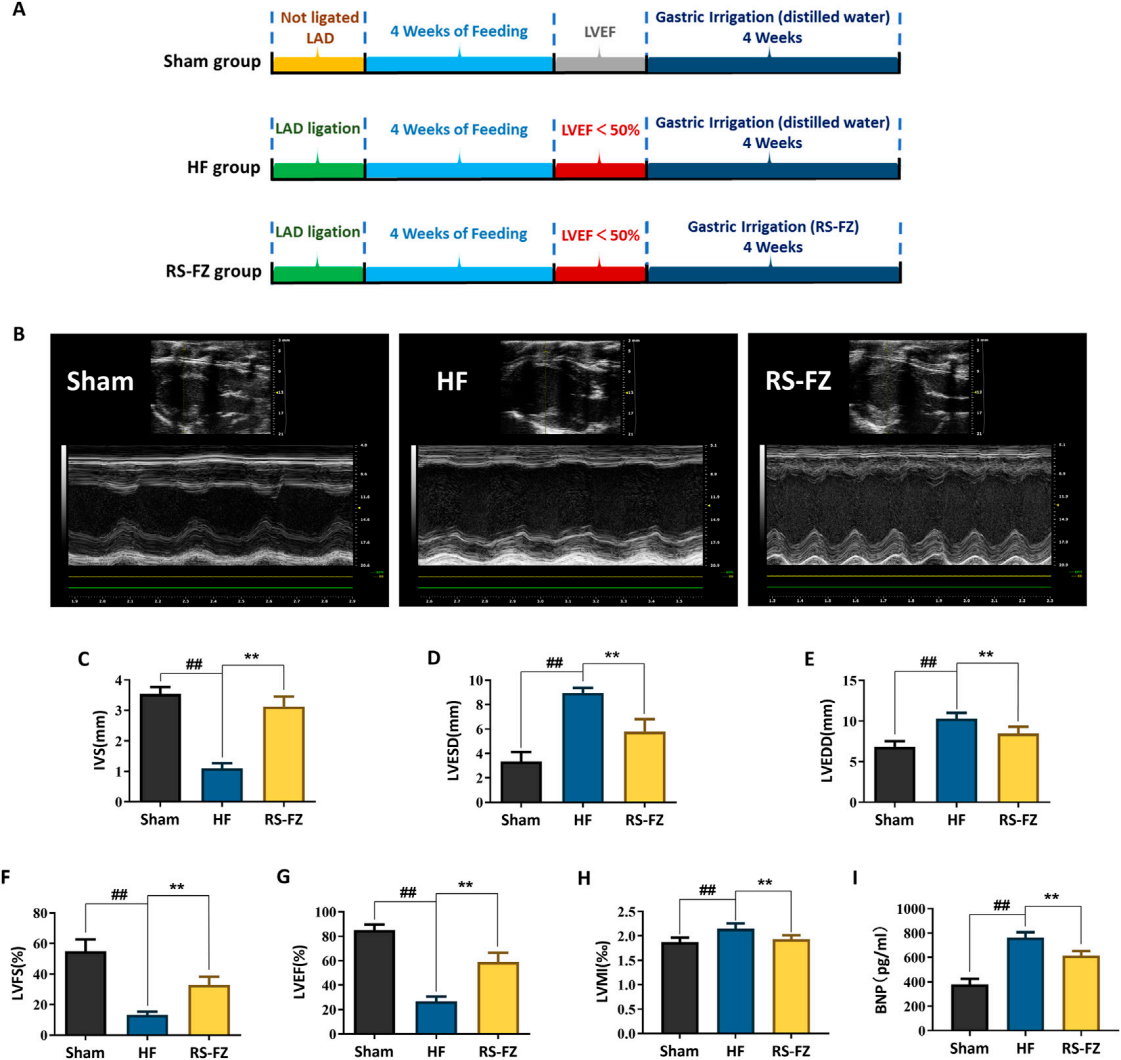
RS-FZ improved cardiac function of rats. **(A)** Experimental steps of HF model; **(B)** Representative M-mode echocardiographic images of Sham, HF model, RS-FZ group rats; **(C)** Ventricular septal thickness (IVS); **(D)** Left ventricular end-diastolic diameter (LVESD); **(E)** Left ventricular end-diastolic diameter (LVEDD); **(F)** Left ventricular short axis shortening (LVFS); **(G)** Left ventricular ejection fraction (LVEF); **(H)** Left ventricular mass index (LVMI); **(I)** The content of brain natriuretic peptide (BNP) in serum. Compared to Sham group, ^##^ *p* < 0.01; Compared to HF model group, **p < 0.01; All data expressed as mean ± SD, *n* = 8/group. Ligation of the anterior descending (LAD).

We evaluated the effect of RS-FZ by constructing an HF model (Figures 2A,B). As shown in Figures 2C–I, compared with the Sham group, LVEDD, LVESD, LVMI, and BNP in the HF group were increased, and IVS, LVEF, and LVFS values were significantly decreased (*p* < 0.01). Compared with the HF group, LVEDD, LVESD, LVMI, and BNP in the RS-FZ group were reduced considerably, and IVS, LVEF, and LVFS were significantly increased (*p* < 0.01). The results suggested that RZ-FZ can significantly ameliorate the cardiac function of HF model rats and significantly reduce the level of diagnostic biomarkers (such as BNP) of HF.

**FIGURE 3:**
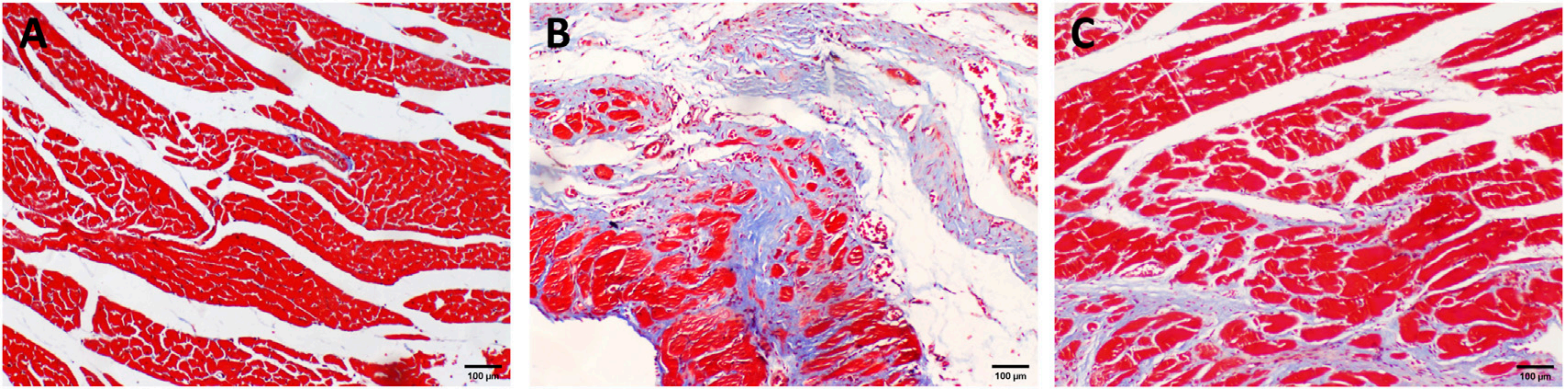
Distribution of collagen in myocardial tissue **(A)** Sham group **(B)** HF group; **(C)** RS-FZ group. Masson staining (scale bar, 100 µm).

As shown in Figure 3, myocardial cells in the Sham group were arranged in bundles with complete structure, and collagen fibers were not stained obviously. The number of myocardial cells in the HF group was significantly reduced, and the arrangement was disordered. Infarct and non-infarct myocardial cells were mixed, collagen fibers were significantly increased, and the network structure was damaged. Compared with the HF group, the collagen in the RS-FZ group increased to varying degrees, and the collagen fiber membrane was incomplete on the transverse section. Morphologically, the RS-FZ group was better than the HF group. These findings were similar to the results of the cardiac ultrasound indexes.

### Multivariate statistical analysis

The mass spectrometer was calibrated with sodium formate, and accuracy was maintained by lock spray using leucine enkephalin as a reference. QC samples could demonstrate the reliability and stability of the UPLC-QTOF/MS system. To correct the drift of sample retention time in the injection process, we used QI software to align and normalize the peaks with QC samples as a reference. Moreover, 15,118 features were extracted in the negative mode, and 8,962 features were extracted in the positive mode.

**FIGURE 4:**
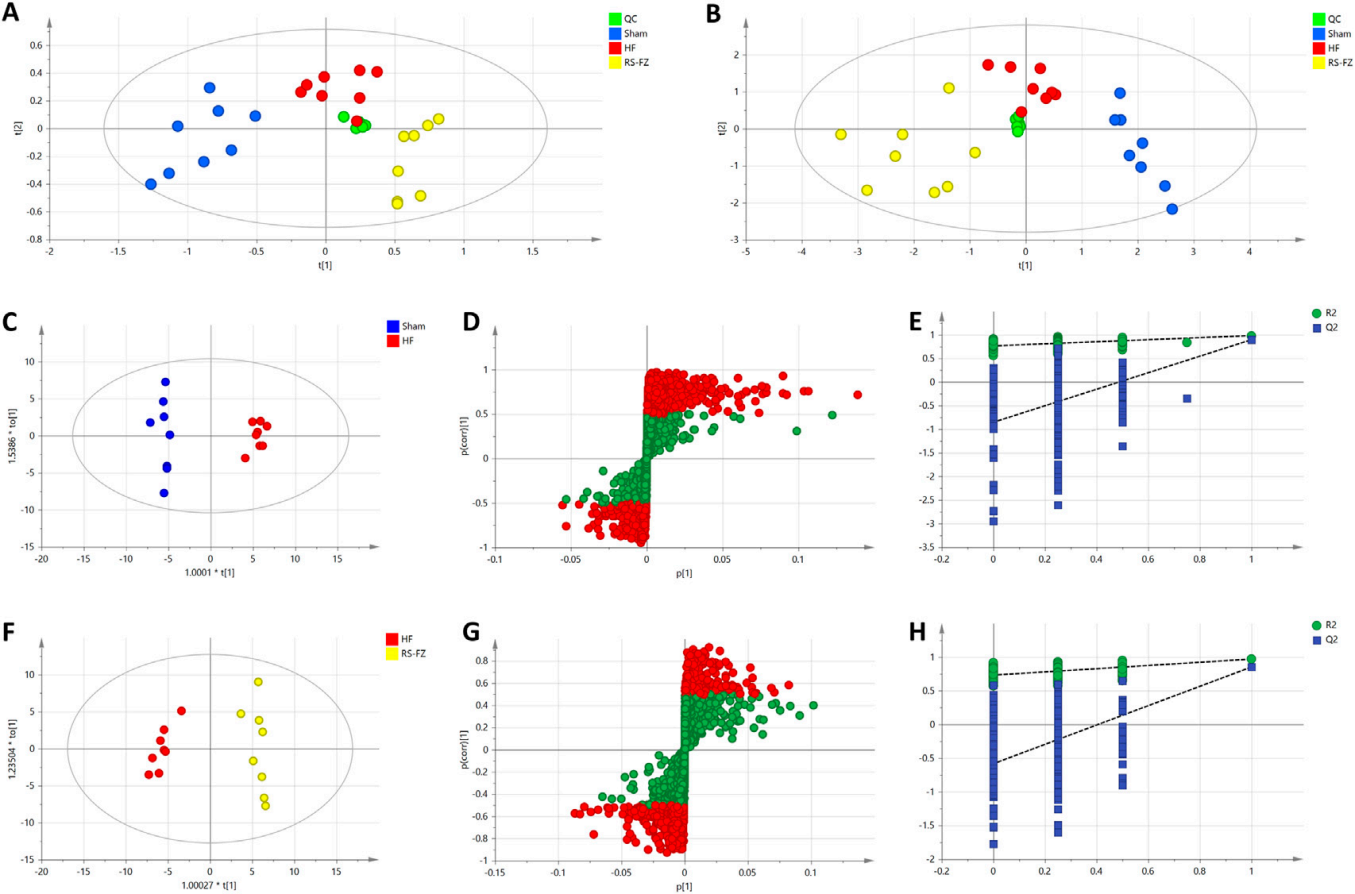
Multivariate statistical analysis results **(A)** PCA score plot of the Sham, HF, and RS-FZ groups in ESI + mode. **(B)** PCA score plot of the Sham, HF, and RS-FZ groups in ESI- mode **(C)** OPLS-DA score plot of Sham and HF groups in ESI- mode **(D)** S-plot of the OPLS-DA model for Sham and HF groups in ESI- mode; **(E)** The 200-permutation test for Sham and HF groups in ESI- mode **(F)** OPLS-DA score plot of HF and RS-FZ groups in ESI- mode; **(G)** S-plot of the OPLS-DA model for HF and RS-FZ groups in ESI- mode **(H)** The 200-permutation test for HF and RS-FZ groups in ESI- mode.

To determine the chemical differences between the different groups of high-dimensional mass spectrometry data, we used PCA and OPLS-DA analysis to evaluate three groups’ and two groups’ metabolomics datasets, respectively. PCA analysis showed that the samples in Sham, HF, and RS-FZ groups could be distinguished. Besides, QC samples (*n* = 6, green circle) were distributed centrally and clustered near the middle of the scoring matrix projection graph, indicating that the UPLC-QTOF/MS system was stable throughout the analysis (Figures 4A,B). The OPLS-DA results showed that the Sham group, HF group, and RS-FZ group were distinguished in both ion modes (Figures 4C,F; Supplementary Figure S1).

In the negative model, R2X (cum) and Q2 (cum) of the PCA model are 0.917 and 0.748. R2X (cum). In addition, R2X (cum), R2Y (cum), and Q2 (cum) of OPLS-DA were 0.906, 0.984, and 0.897 by comparing the Sham group and HF group, respectively. R2X (cum), R2Y (cum), and Q2 (cum) of OPLS-DA were 0.818, 0.971, and 0.853 by comparing the HF group and RS-FZ group, respectively. The analysis of the above parameters shows that both PCA and OPLS-DA models have good quality and accuracy.

In the negative (Figures 4D,G) and positive (Supplementary Figure S1) models, red-labeled variables (|p(corr)| ≥ 0.5) were considered to be more conducive to the separation between different experimental groups in the S-plot analysis. Permutation tests with 200 iterations were performed to verify the validity of the data multivariate analysis model. The results indicated that the original models are valid (Figures 4E,H).

### Identification of specific metabolites of RS-FZ for heart failure

**FIGURE 5:**
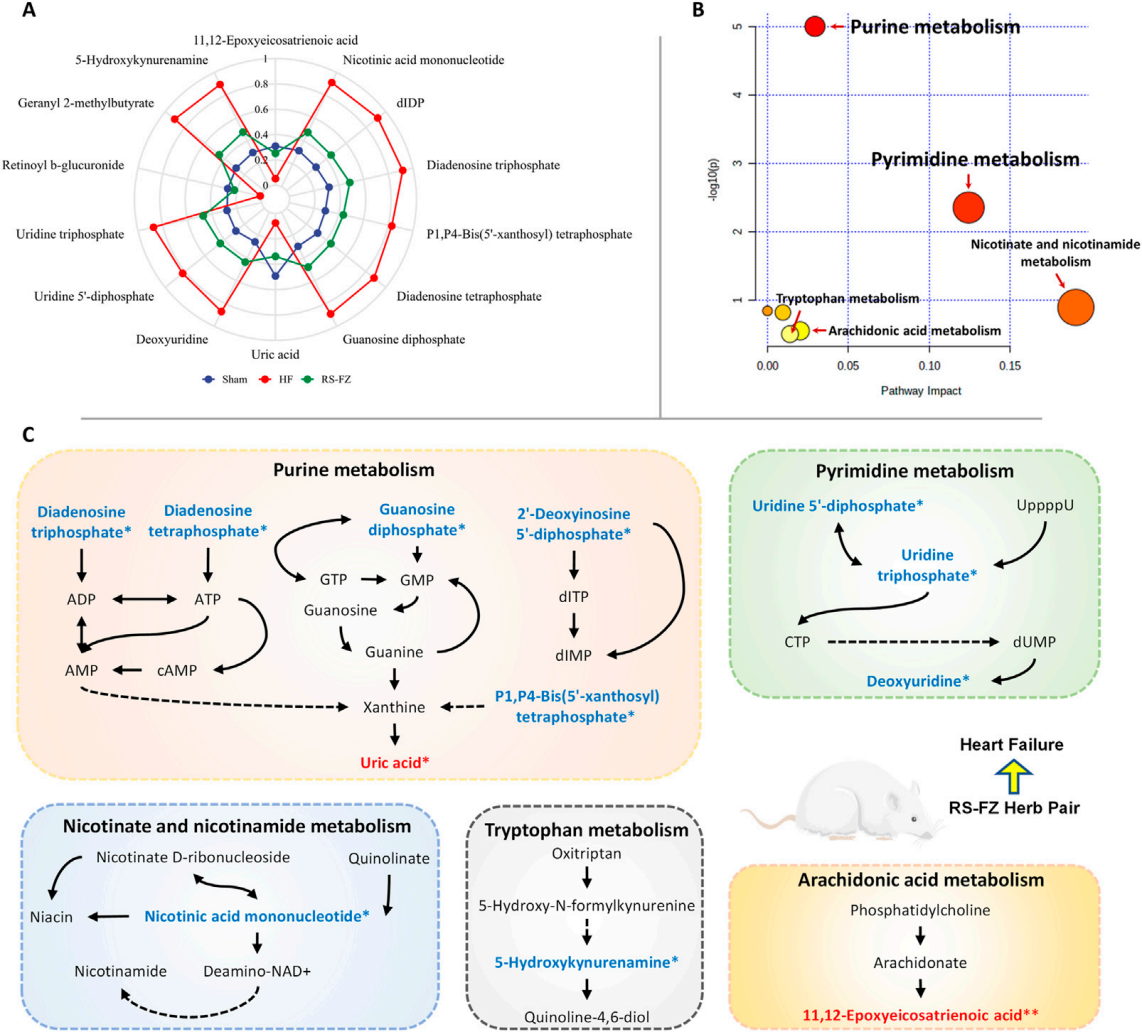
Overall metabolic profile **(A)** Radar chart of relative abundance of 14 specific metabolites **(B)** Metabolic pathways as visualized using MetaboAnalyst **(C)** Representative metabolic pathways demonstrated in metabonomics analysis, blue represents metabolites downregulated by RS-FZ whereas red represents metabolites up-regulated by RS-FZ.

OPLS-DA analysis revealed endogenous metabolites with *p* < 0.05, VIP >1, and |p(corr)| ≥ 0.5 from Sham, HF, and RS-FZ groups in the positive and negative modes that were screened as potential biomarkers. By searching and matching HMDB metabolic database, KEGG online database, and literature information method, 14 specific metabolites were identified (Table 1; Supplementary Figure S2; Figure 5A). Compared with the Sham group, 11 metabolites were significantly up-regulated in the HF group; three other metabolites were significantly downregulated. After treatment, these specific metabolites in the RS-FZ group recovered to varying degrees.

**TABLE 1:** Metabolites identified as potential biomarkers.

RT (min)	Quasi-molecular ion (m/z)	Mass error (ppm)	KEGG ID	Chemical name	HF vs. sham		HF vs. RS-FZ	
					VIP	FC	VIP	FC
*0.89	313.0410 [M-H]-	−5.08	C00341	Geranyl diphosphate	3.03	3.08	1.51	1.59
*0.86	335.0691 [M-H]-	3.84	C01185	Nicotinic acid mononucleotide	9.87	2.83	1.53	1.66
*10.00	319.2274 [M-H]-	−1.48	C14770	11,12-Epoxyeicosatrienoic acid	2.13	0.65	4.50	0.43
*0.93	755.0681 [M-H]-	−2.57	C06197	Diadenosine triphosphate	2.72	2.98	1.53	1.94
*0.87	229.0725 [M + H]+	−0.40	C00526	Deoxyuridine	12.97	3.11	1.50	1.80
0.85	411.0052 [M-H]-	−2.46	C01344	2′-Deoxyinosine 5′-diphosphate	1.99	2.86	1.49	1.93
0.99	835.0182 [M-H]-	−5.58	C01260	Diadenosine tetraphosphate	3.80	2.85	1.51	2.18
*1.08	442.0064 [M-H]-	−3.31	C00035	Guanosine diphosphate	1.94	2.83	1.55	1.73
*0.88	181.0851 [M + H]+	−0.90	C05638	5-Hydroxykynurenamine	2.49	2.81	1.53	2.04
*0.91	405.0097 [M + H]+	1.39	C00015	Uridine 5′-diphosphate	2.46	2.83	1.54	2.05
0.96	869.0005 [M-H]-	−1.03	C04392	P1,P4-Bis(5′-xanthosyl) tetraphosphate	2.82	3.07	1.53	1.95
*9.24	475.2364 [M-H]-	−0.86	C11061	Retinoyl b-glucuronide	1.55	0.64	11.42	0.60
*1.17	483.0149 [M-H]-	8.59	C00075	Uridine triphosphate	6.60	2.18	1.54	1.81
*1.30	167.0201 [M-H]-	−1.51	C00366	Uric acid	3.66	0.56	2.35	0.63

RT, run time; * had detected fragment ions shown in Figure S2; VIP, variable important in the projection; FC, fold change.

### Metabolic pathway enrichment analysis

Herein, 14 potential metabolites were expressed at significant levels. Pathway analysis was performed in detail using Metaboanalyst (Figure 5B). The results showed that the screened specific metabolites were related to Purine, Pyrimidine, Nicotinate and nicotinamide, Arachidonic acid, and Tryptophan metabolism pathways. The main metabolic pathways are shown in Figure 5C.

### “Metabolite-target-component” interactive network and analysis

**FIGURE 6:**
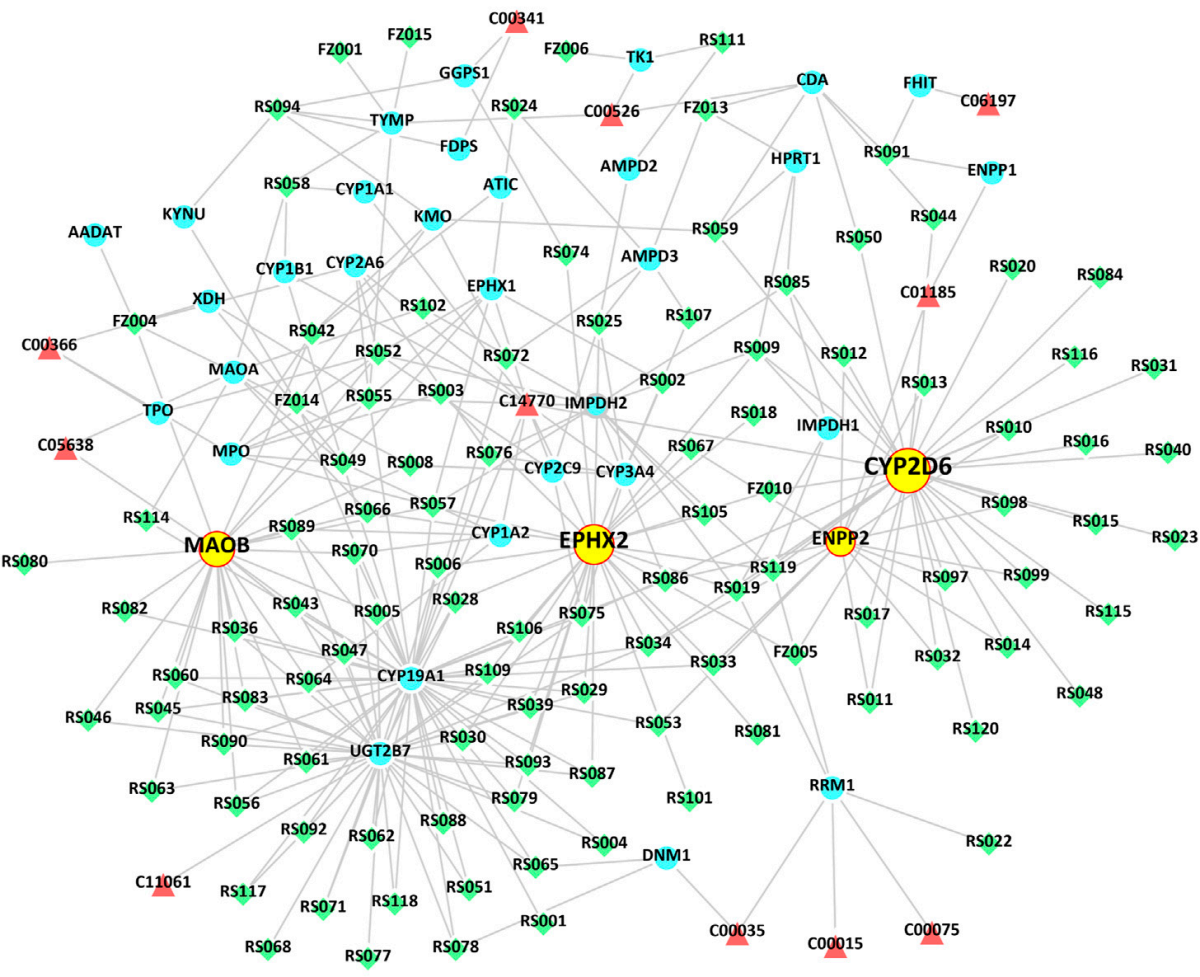
The “Metabolite-Target-Component” interactive network participates in the treatment of HF by RS-FZ. The green diamonds represent active components of RS-FZ. The red triangles represent the specific metabolites. The blue dots represent common targets of RS-FZ components and specific metabolites (Yellow dots represent the critical targets that have been validated by immunohistochemistry).

We collected 218 related target proteins of the specific metabolites by MBROLE database and analyzed the target proteins of RS-FZ (Supplementary Table S1) affecting the level of endogenous substances by network pharmacology prediction. The M-T-C interactive network is constructed and analyzed by Cytoscape 3.8.2 software. There were 11 specific metabolites, 35 intersection target proteins, and 108 compounds of TCM involved in the network **(** Figure 6; Supplementary Table S2). Furthermore, RS-FZ might directly regulate 11 downstream specific metabolites by acting on these 35 target proteins.

### Effect of RS-FZ on the expression of critical direct targets associated with the treatment of HF

The first four critical target proteins of RS-FZ regulating downstream specific metabolites were screened through the M-T-C interactive network topology analysis. The results showed (Table 2) that most compounds of RS-FZ mainly acted on CYP2D6 (P10635; degree = 33), EPHX2 (P34913; degree = 31), MAOB (P27338; degree = 25), and ENPP2 (Q13822; degree = 16) directly regulated the three specific metabolites, 11,12-Epoxyeicosatrienoic acid (C14770), 5-Hydroxykynurenamine (C05638) and Nicotinic acid mononucleotide (C01185). These results indicate that these essential target proteins are affected by multiple components of RS-FZ and then regulate the changes of downstream specific metabolites.

**TABLE 2:** The critical direct targets of RS-FZ for HF.

No.	UniProt ID	Protein name	Gene name
1	P10635	Cytochrome P450 2D6	CYP2D6
2	P34913	Soluble epoxide hydrolase 2	EPHX2
3	P27338	Monoamine oxidase-B	MAOB
4	Q13822	Ectonucleotide Pyrophosphatase/Phosphodiesterase 2	ENPP2

**FIGURE 7:**
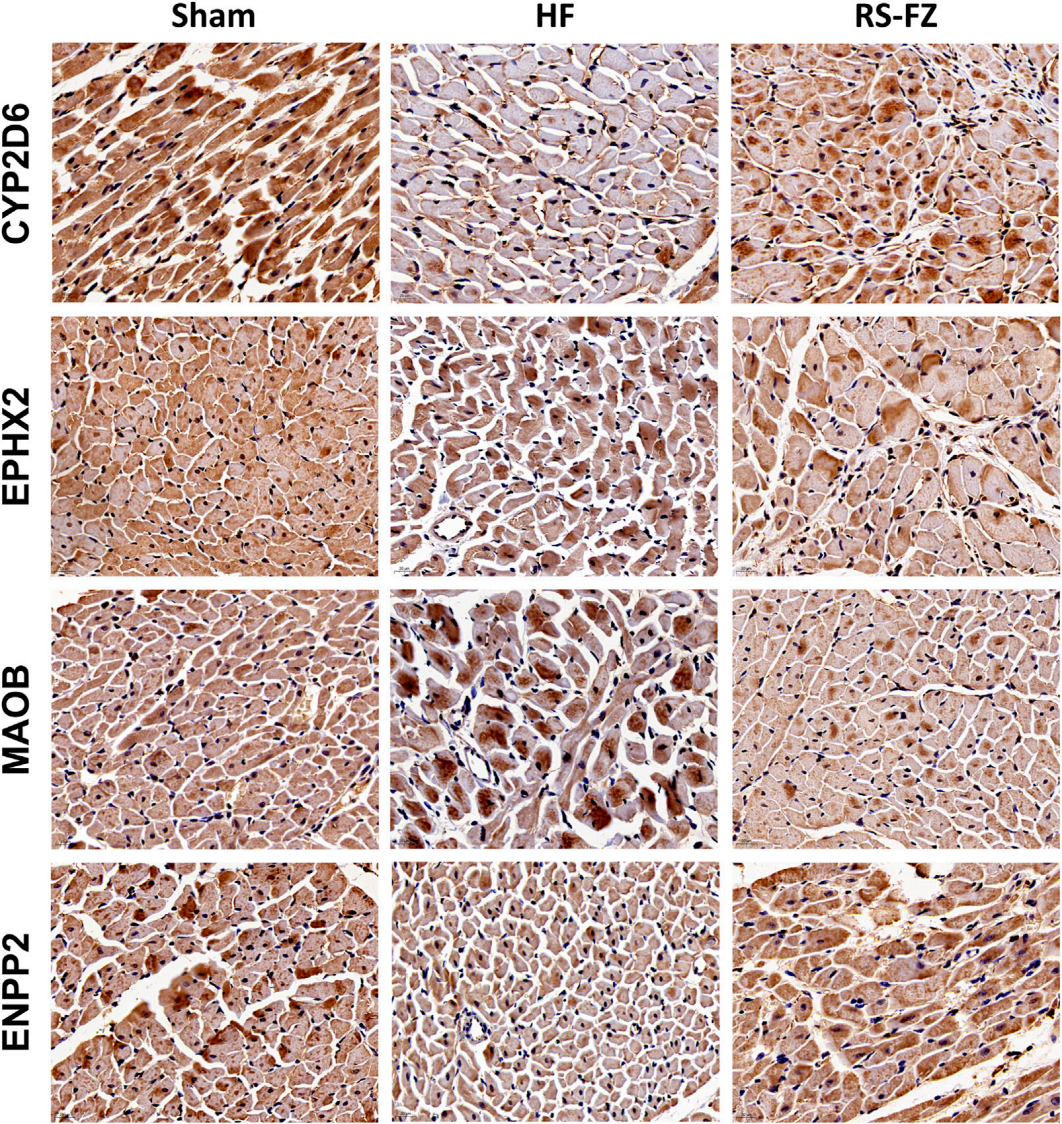
Effect of RS-FZ on the expression of the critical targets in the treatment of HF detected by immunohistochemistry staining (×400). The vital targets detected in heart tissue are CYP2D6, EPHX2, MAOB, and ENPP2.

**FIGURE 8:**
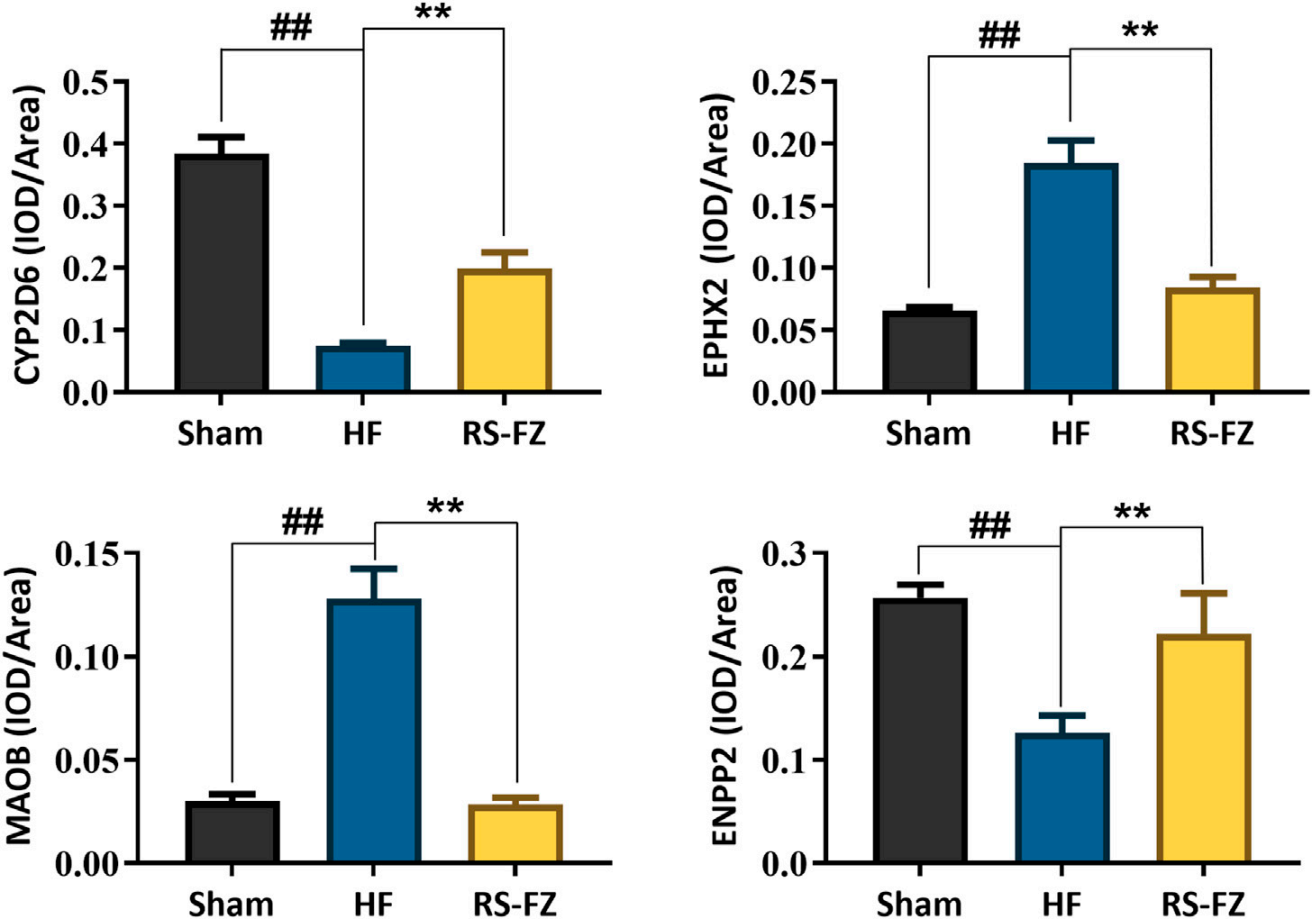
The effect of RS-FZ on the expression of CYP2D6, EPHX2, MAOB, and ENPP2 in the treatment of HF detected by immunohistochemistry staining (×400). The data are expressed as the mean ± SD, *n* = 3. Compared to Sham group, ^##^ *p* < 0.01; Compared to HF model group, *p < 0.05, **p < 0.01.

To continue to explore the significance of the biological mechanism of the critical target proteins mentioned above and verify the authenticity of the interactive network analysis, we used immunohistochemical techniques to detect the effects of RS-FZ on the expression of CYP2D6, EPHX2, MAOB, and ENPP2 proteins. As shown in Figures 7, 8, compared with the Sham group, the expression levels of CYP2D6 and ENPP2 proteins in the HF group were significantly decreased. The expression of EPHX2 and MAOB proteins increased significantly. These results suggest that the expression levels of these four target proteins in the heart of rats are statistically significant, and compared with the HF group, RS-FZ had a good intervention effect. Therefore, we believe that CYP2D6, EPHX2, MAOB, and ENPP2 are the critical target proteins of specific metabolites regulated by RS-FZ.

## Discussion

An abundance of studies has shown that RS and FZ strengthen the heart, anti-inflammatory, cardiovascular protection, and other intervention and treatment of HF pharmacological effects (Wang et al., 2021; Wang et al., 2020; Zhang and Li, 2013). Combined with the results of pharmacodynamics, we found that RS-FZ significantly ameliorated myocardial fibrosis, enhanced cardiac function, and reduced the serum HF marker (BNP) level in rats with HF, with an apparent therapeutic effect. However, the molecular mechanism of RS-FZ has not been revealed in the actual metabolic environment. In this study, the specific metabolites and metabolic pathways of RS-FZ intervention in HF rats were screened based on metabonomics in Figure 5. Research shows that these metabolic pathways promote angiogenesis, anti-fibrosis, anti-apoptosis, heart protection, energy homeostasis, and anti-inflammatory regulation (Camici et al., 2018; Chen et al., 2020; De Abreu et al., 2003; Hyde and Missailidis, 2009; Imura et al., 2002; Ma et al., 2016). In conclusion, compared to the HF model group, RS-FZ treatment in HF rats improved various biological functions regulated by different metabolic pathways. However, the upstream signal targets regulating the above specific metabolites are unclear.

Network pharmacological analysis methods have been increasingly used in drug development and molecular mechanisms discovery. This method can also further evaluate specific metabolite-related targets regulated by RS-FZ. Through the analysis of the M-T-C interaction network, we screened out four essential target proteins (CYP2D6, EPHX2, MAOB, and ENPP2) that RS-FZ interferes with the change of specific metabolite content.

### Identified compounds

Among the 79 small molecule compounds that RS-FZ might act on these four critical target proteins, salsolinol, a plant-based isoquinoline alkaloid, was originally isolated from FZ and identified as an active cardiotonic component of FZ. The research reported that salsolinol-alleviated doxorubicin-induced chronic heart failure (CHF) in rats by improving cardiac function, reducing serum levels of myocardial injury markers, alleviating cardiac tissue damage, and increasing the relative mRNA expression levels of key enzymes downstream of the tricarboxylic acid cycle *in vivo*, thereby enhancing myocardial energy metabolism (Wen et al., 2019). Salsolinol also acts as an inhibitor of MAOB by interfering with the catalytic process of monoamine oxidase for the oxidative deamination of biogenic and xenobiotic amines (Strolin et al., 1987; Patsenka et al., 2004). Benzoylmesaconine is the most abundant monoester alkaloid of Aconitum species, and can significantly inhibit the release of proinflammatory cytokines and mediators in lipopolysaccharide (LPS) activated RAW264.7 macrophages and has good anti-inflammatory effects (Zhou et al., 2022). Beiwutine is a nortriterpenoid alkaloid isolated from Aconitum kusnezoffii together, and evidence from studies has shown that Aconitum alkaloids at safe doses have cardiotonic, increased cardiac contraction frequency, elevated blood pressure, and increased peripheral blood flow, among other pharmacological effects, and have promising therapeutic potential in HF (Ishimi et al., 2006; Li et al., 2022). Ginsenoside Rb1 (Rb1), as one of the main chemical components of ginseng, has been found *in vivo* to attenuate angiotensin II-induced cardiac hypertrophy, cardiac inflammation as well as systemic inflammation. In cardiomyocytes, Rb1 directly counteracts the pro-hypertrophic effects of angiotensin II and phenylephrine and maintains mitochondrial function. In LPS-stimulated macrophages, Rb1 reduced proinflammatory mediators of inflammation, such as interleukin (IL)-1β, IL-6, and tumor necrosis factor (TNF), thereby exerting anti-inflammatory activity (Wang et al., 2021a; Zheng et al., 2017). Ginsenoside Rg2 reduces the expression of fibrosis-associated genes collagen I, collagen III, and collagen IV by activating angiotensin II-induced phosphorylated AKT in cardiac fibroblasts α-Smooth muscle actin, thereby improving cardiac function and attenuating cardiac fibrosis (Li et al., 2020). In addition, based on studies showing that incubation of ginsenoside Rb1, Rb2, Rc, Rd, Re, Rf, and Rg1 with a panel of recombinant human CYP450 isoforms revealed that ginsenoside Rd exerted a little inhibitory effect on alternative substrates of the recombinant enzyme CYP2D6, that ginsenoside Rc enhanced the activity of the recombinant enzyme CYP2C9, and that ginsenoside Rf increased the activity of the recombinant enzyme CYP3A4 (Henderson et al., 1999). Salicylic acid and its derivatives have various biological activities, such as acting as MAOB inhibitors, anti-inflammation, and antioxidation (Benny et al., 2022). In summary, based on the M-T-C interaction network, we found that RS-FZ treatment of HF may be due to the modulation of CYP2D6, EPHX2, MAOB, and ENPP2 by a variety of small molecules with potent cardiac, anti-inflammatory, anti-fibrotic and other pharmacological effects.

### CYP2D6

CYP2D6 is a cytochrome P450 monooxygenase involved in the metabolism of fatty acids, steroids, and retinoids. Also involved in the oxidative metabolism of drugs such as anti-HF and adrenoceptor antagonists. Among its related pathways are glucose/energy Metabolism and oxidation by cytochrome P450 (Klotz, 2007). The purified ginseng dry extract inhibited or induced human liver CYP450 *in vitro*. The results showed that the ginseng dry extract did not affect the metabolic activity of recombinant CYP2D6 and could not mediate clinically significant drug-drug interactions (Zheng et al., 2014). In addition, arachidonic acid can be metabolized by recombinant CYP2D6 to 5,6-, 8,9-, 11,12-, and 14,15-epoxyeicosatrienoic acid (EET) with anti-inflammation, anti-fibrosis, and anti-apoptosis effects (Li et al., 2021; Snider et al., 2008). In this study, we indeed found that RS-FZ could increase the metabolite levels of 11,12-EET in HF rats based on metabolomics.

### EPHX2

EPHX2 is a member of the epoxide hydrolase family. Proteins found in the cytoplasm and peroxisomes bind to specific epoxides and convert them into corresponding dihydrodiols (Newman et al., 2003). Studies have shown that EPHX2 can hydrolyze endogenous anti-inflammatory mediator EETs into less active diols (Gowler et al., 2022). However, EETs can reduce inflammation, regulate endothelial tension, improve mitochondrial function, reduce oxidative stress, and activate various stress response systems, such as the phosphoinositol 3-kinase (PI3K) pathway during cardiac ischemia-reperfusion injury, enhancing membrane ion channel activity, and improving functional recovery after left ventricular ischemia (Chaudhary et al., 2009). Therefore, inhibition of EPHX2 to maintain high EET levels is considered to be a new target for clinical application in the treatment of cardiovascular diseases (Turanli et al., 2022). Based on the role of EPHX2 in HF in knockout mice, it was found that EPHX2 gene ablation could prevent HF and arrhythmia caused by pressure overload (Monti et al., 2008).

### MAOB

MAOB, as a form of monoamine oxidase (MAO), catalyzes the oxidative deamination of biogenic amines and allogenic amines (Hassan et al., 2022). In wild-type mice, pressure overload induced by transverse aortic constriction (TAC) leads to enhanced dopamine catabolism, left ventricular (LV) remodeling, and dysfunction. In contrast, mice deficient in MAOB that received TAC preserved LV function at both early and late stages. Furthermore, in neonatal and adult cardiomyocytes, enhanced MAO activation triggers oxidative stress with a decline in mitochondrial membrane potential when the ATP synthase inhibitor oligomycin is present. But the MAOB inhibitor pargyline completely counteracted this change, suggesting that MAO activation induces underlying mitochondrial dysfunction and favors the pathogenesis of HF (Kaludercic et al., 2014).

### ENPP2

ENPP2 is both a phosphodiesterase, which cleaves phosphodiester bonds at the 5′ end of oligonucleotides, and a phospholipase, which catalyzes the production of lysophosphatidic acid (LPA) in extracellular fluids. LPA evokes growth factor-like responses including stimulation of cell proliferation and chemotaxis (Borza et al., 2022). ENPP2, as a lipid kinase involved in lipid metabolism, is protective against erastin-induced cardiomyocyte iron death. The classical ferroptosis inducer erastin remarkably inhibits the growth which could be rescued by the small molecule Fer-1 in H9c2 cells. Adenovirus-mediated ENPP2 overexpression modestly promoted H9c2 cell migration and proliferation and significantly inhibited erastin-induced H9c2 cell iron death (Bai et al., 2018; He et al., 2021). It is suggested that RS-FZ may promote cardiomyocyte survival and proliferation perhaps through this target.

The above findings show that RS-FZ intervention in HF rats mainly acted on CYP2D6, EPHX2, MAOB, and ENPP2 proteins, thereby might improving inflammatory response, cardiac fibrosis, apoptosis, and mitochondrial dysfunction. However, these four targets were identified only by computational analysis, and the results need to be further validated by animal experiments. Therefore, we used immunohistochemical techniques to investigate the effects of RS-FZ on the expression of these four target proteins in the heart of HF rats. The results showed that CYP2D6 and ENPP2 proteins decreased significantly, and EPHX2 and MAOB proteins increased substantially in HF rats. RS-FZ significantly reversed the expression of crucial proteins in HF rats. This also supports the possible mechanism of RS-FZ in the treatment of HF.

## Conclusion

Our study has proved that RS-FZ significantly ameliorated myocardial fibrosis, enhanced cardiac function, and reduced the serum HF marker (BNP) level in rats with HF, with an apparent therapeutic effect. Moreover, the combination of network pharmacology and serum metabolism reveals that RS-FZ plays a multi-level and multi-faceted role in treating HF. Of course, as an early-stage exploratory study, we are complying with the 4R principles (reduction, replacement, refinement, and responsibility). Thus, this study must be viewed in light of its strengths and limitations, many of which represent opportunities for future research.

## Data Availability

The raw data supporting the conclusions of this article will be made available by the authors, without undue reservation.
