# Supplementary material for: The mechanism of Renshen-Fuzi herb pair for treating heart failure—Integrating a cardiovascular pharmacological assessment with serum metabolomics
Source: Front Pharmacol. 2022 Dec 5;13:995796. doi: 10.3389/fphar.2022.995796 (PMC9760753; doi:10.3389/fphar.2022.995796)
Supplement: Supplementary file 2 [file Image2.pdf]

KEGG ID: C00341

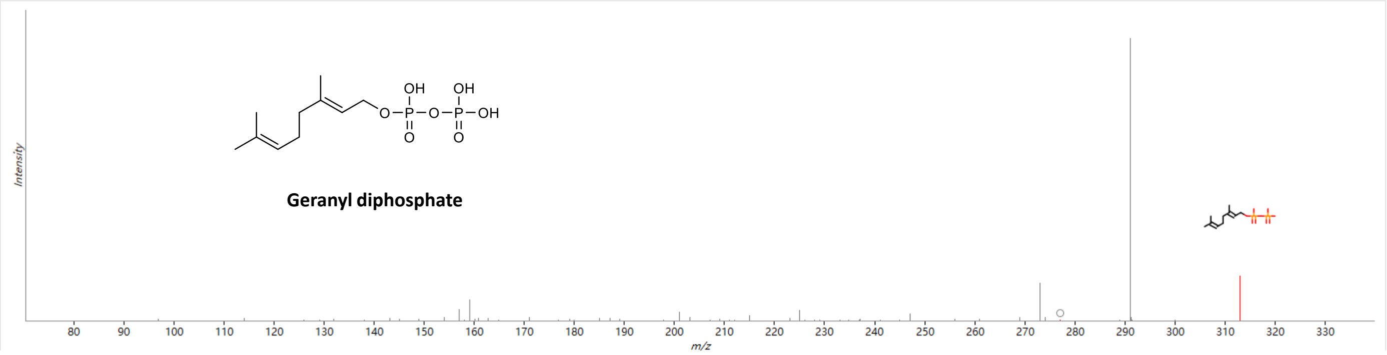

KEGG ID: C14770

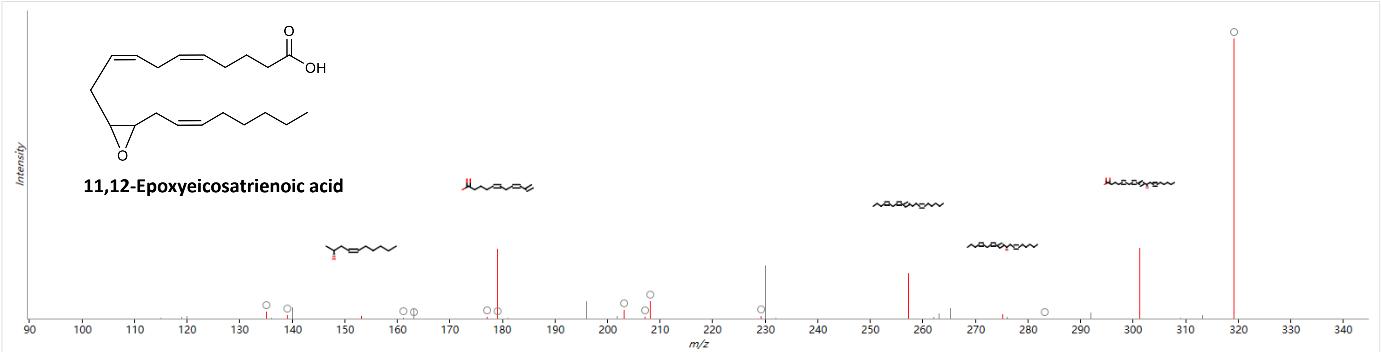

KEGG ID: C06197

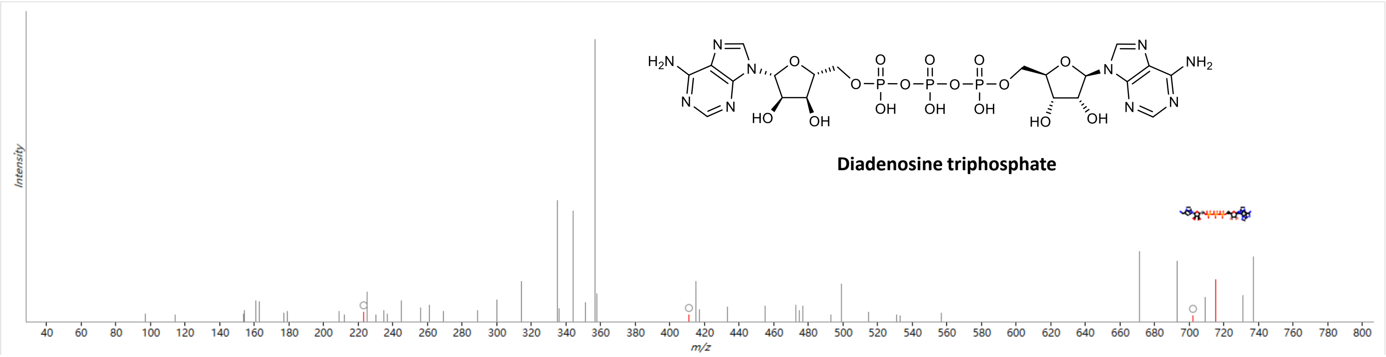

KEGG ID: C00526

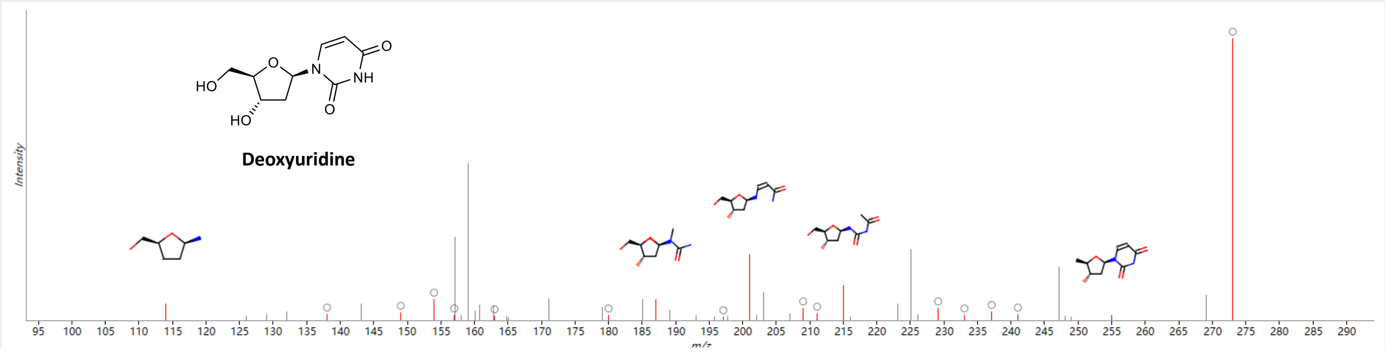

KEGG ID: C01260

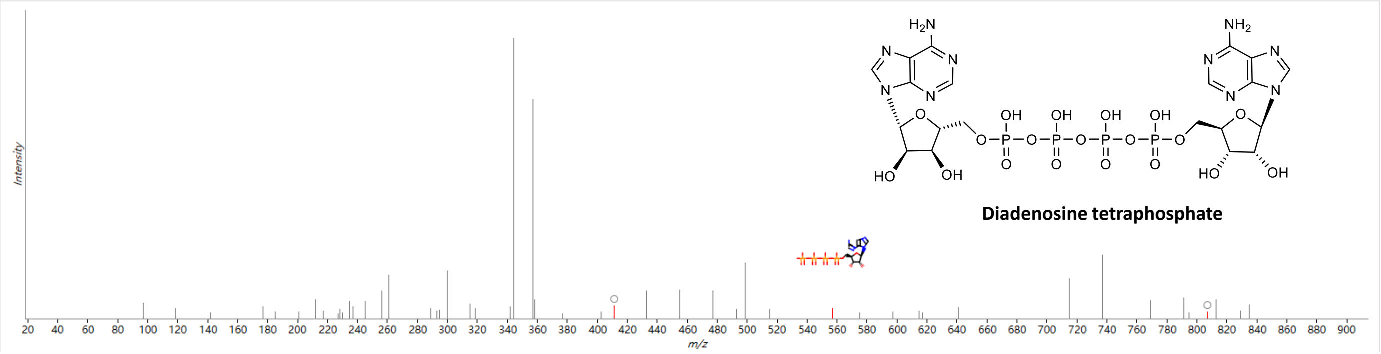

KEGG ID: C00035

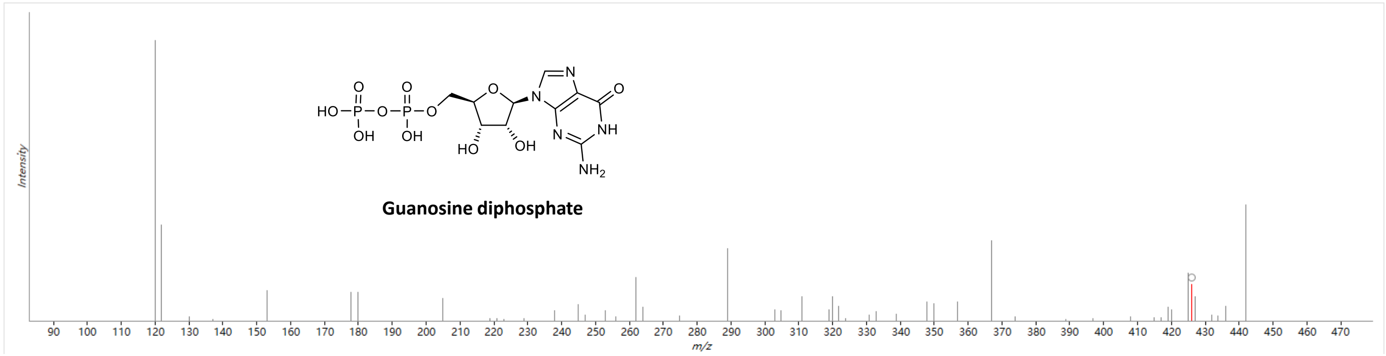

KEGG ID: C05638

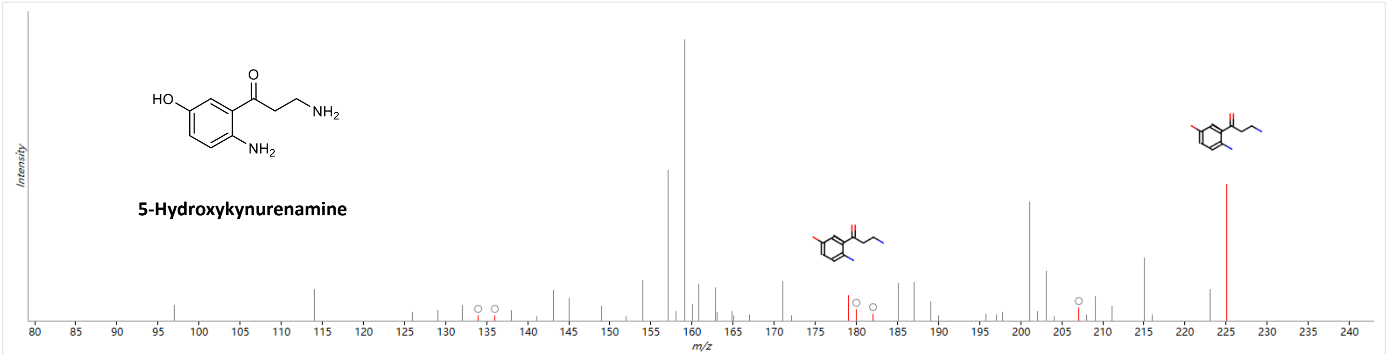

KEGG ID: C00015

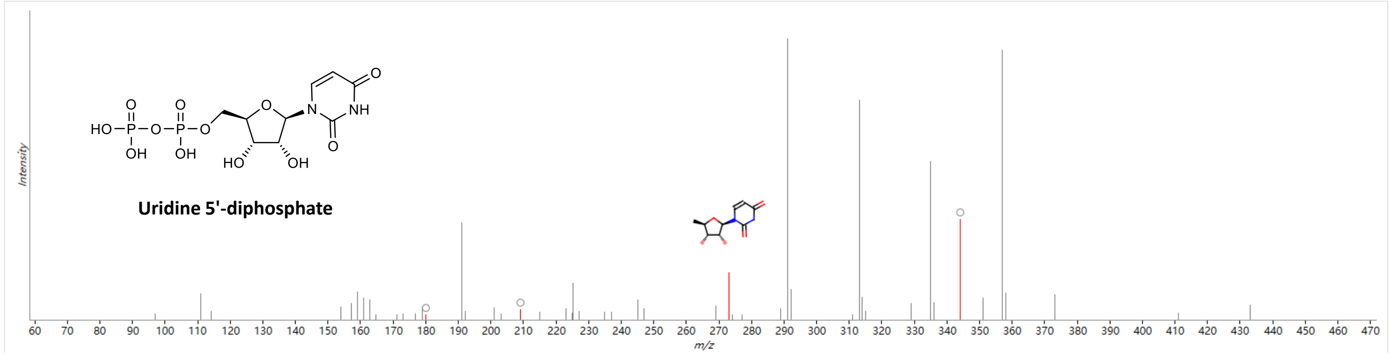

KEGG ID: C04392

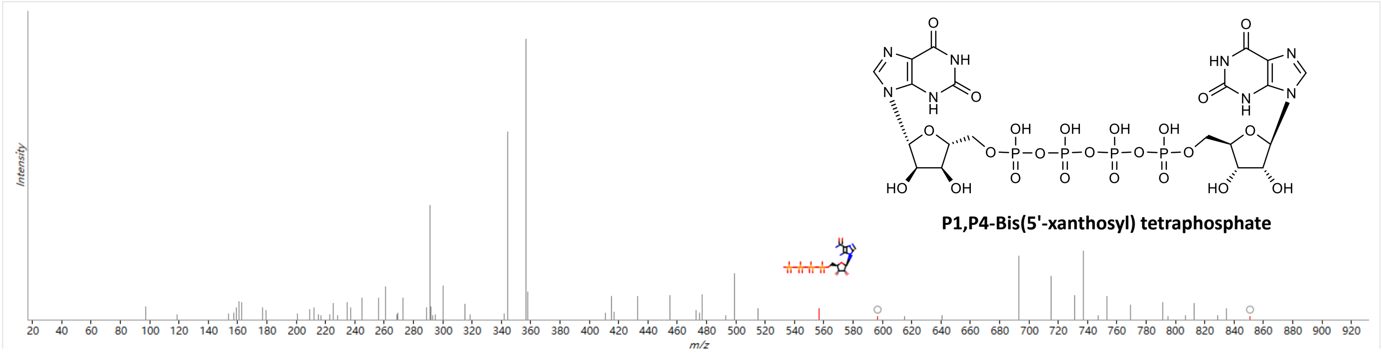

KEGG ID: C11061

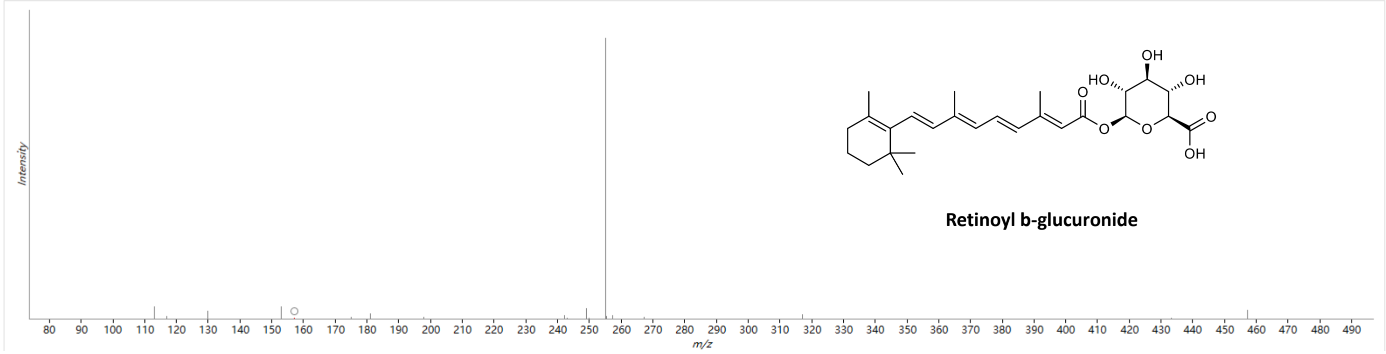

KEGG ID: C00366

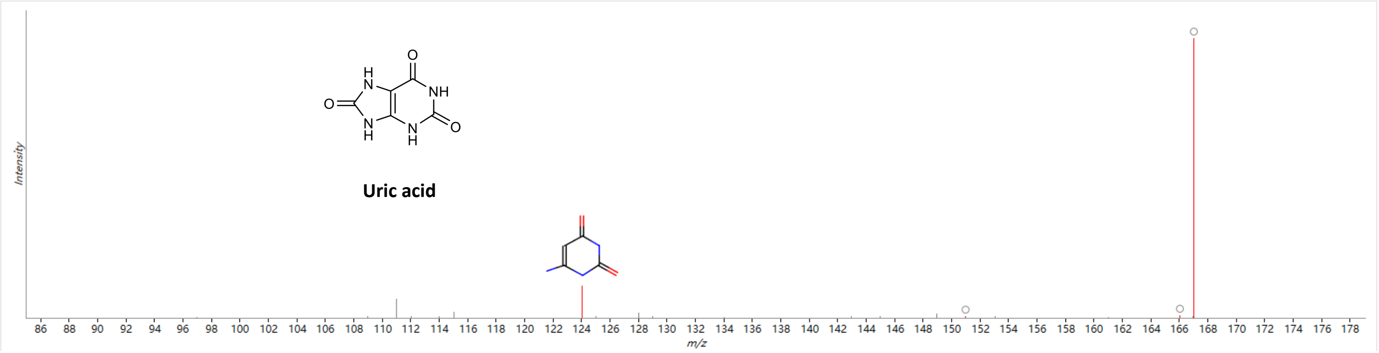

Supplementary Figure S2 | The 11 metabolites and their fragment ions detected by UPLC-QTOF/MS analysis.
