# Supplementary material for: The mechanism of Renshen-Fuzi herb pair for treating heart failure—Integrating a cardiovascular pharmacological assessment with serum metabolomics
Source: Front Pharmacol. 2022 Dec 5;13:995796. doi: 10.3389/fphar.2022.995796 (PMC9760753; doi:10.3389/fphar.2022.995796)
Supplement: Supplementary file 4 [file Image1.pdf]

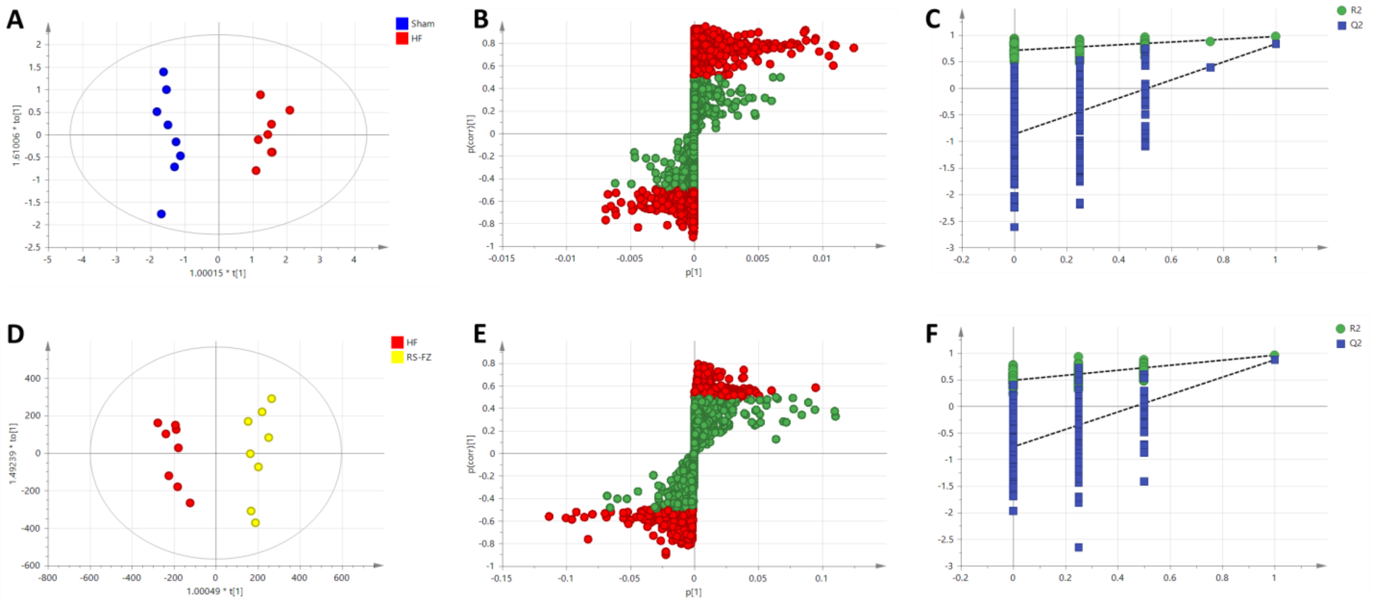

Supplementary Figure S1 | (A) OPLS-DA score plot of Sham and HF groups in ESI+ mode; **(B)** S-plot of the OPLS-DA model for Sham and HF groups in ESI+ mode; **(C)** The 200-permutation test for Sham and HF groups in ESI+ mode; **(D)** OPLS-DA score plot of HF and RS-FZ groups in ESI+ mode; **(E)** S-plot of the OPLS-DA model for HF and RS-FZ groups in ESI+ mode; **(F)** The 200-permutation test for HF and RS-FZ groups in ESI+ mode.
